# State Attachment Variability: Between- and within-Person Level Associations with Trait Attachment and Psychological Problems

**DOI:** 10.3390/brainsci11101264

**Published:** 2021-09-24

**Authors:** Martine W. F. T. Verhees, Eva Ceulemans, Marian J. Bakermans-Kranenburg, Guy Bosmans

**Affiliations:** 1 Clinical Psychology, KU Leuven, Tiensestraat 102, 3000 Leuven, Belgium; guy.bosmans@kuleuven.be; 2 Quantitative Psychology and Individual Differences, KU Leuven, Tiensestraat 102, 3000 Leuven, Belgium; eva.ceulemans@kuleuven.be; 3 Centre for Child and Family Studies, Vrije Universiteit Amsterdam, Van der Boechorststraat 7, 1081 BT Amsterdam, The Netherlands; m.j.bakermans@vu.nl

**Keywords:** attachment, intra-individual variability, state attachment, psychological problems, middle childhood

## Abstract

Research suggests that inter-individual differences in the degree of state attachment variability are related to differences in trait attachment and psychological problems between children. In this study, we tested whether such associations are also relevant at a within-person level, and if so, whether intra-individual fluctuations in the degree of variability were predictive of or predicted by intra-individual fluctuations in trait attachment and psychological problems. Children (*N* = 152; M_age_ = 10.41 years, SD_age_ = 0.60 at time 1) were tested three times over a period of one year. At each timepoint, children reported on their expectations of maternal support in different distressing situations. Additionally, we administered measures of trait attachment to children and psychological problems to children and their mothers. We used Random-Intercept Cross-Lagged Panel Models to distinguish between-person from within-person associations between these constructs over time. The results revealed that the degree of state attachment variability was mainly relevant to understand differences between children in trait attachment and psychological problems: children who overall showed more state attachment variability were overall less securely attached at a trait-level and reported more psychological problems. Although evidence for within-person associations was less robust, there was some indication that the degree of state attachment variability might be related to the development of trust and psychological problems at a within-person level.

## 1. Introduction

Ample research has shown that children’s attachment security is linked to their psychological functioning [1]. Recent studies suggest, however, that attachment can best be understood as comprising both a stable trait-like component as well as a more dynamic state component that reflects context-specific attachment expectations [2,3]. Interestingly, it seems that the degree in which children intra-individually vary in their state attachment reflects an inter-individual difference factor that is associated with trait attachment, such that children who are more securely attached at a trait level seem to vary less in their state attachment [2,4]. Moreover, the degree of state attachment variability has been found to explain inter-individual differences in psychological problems, over and above trait attachment [4]. However, due to their cross-sectional designs, previous studies could not test whether such associations are also relevant at a within-person level, i.e., whether intra-individual changes in the degree of state attachment variability contribute to intra-individual changes in trait attachment and psychological problems or vice versa.

It is important to distinguish between-level from within-level associations, as relations found at a between-subjects level may not appropriately describe within-person processes [5]. That is, an association found at a between-person level may not be relevant to explain intra-individual variation or may reflect a different process at a within-person level, which may even lead to opposing associations at a between-person and within-person level [6]. A hypothetical example for the association between the degree of state attachment variability and psychological problems is as follows: it may be that, across children, less variability in attachment states is related to less psychological problems, thus pointing to a positive association at a between-person level (this may reflect adaptive stability of trust in caregiver support). However, at a within-person level, an intra-individual (short-term) decrease in variability may reflect increased (maladaptive) rigidity and predict intra-individual increases in psychological problems, resulting in a negative association at a within-person level.

The current study aimed to explore whether the degree of state attachment variability is only a between-level correlate of trait attachment and psychological problems, or whether the degree of state attachment variability represents a process related to within-person changes in trait attachment and psychological problems, and if so, what predicts what. To address this aim, we used a longitudinal design and Random Intercept Cross-Lagged Panel Modeling (RI-CLPM) [7] to disentangle between-person and within-person level associations between the degree of state attachment variability and (1) trait attachment, and (2) psychological problems.

### 1.1. Trait and State Attachment

Attachment is commonly conceptualized as a relatively stable, trait-like feature after the first five years [8]. When children consistently experience their caregivers to sensitively support them to explore the world and regulate distress, they are proposed to become more securely attached at a trait level [8,9]. Central to secure trait attachment are expectancies that reflect trust in caregiver support during distress. Such expectancies are proposed to be structured in a cognitive script: the secure base script [10]. The secure base script summarizes expectations of caregiver support across script-relevant, i.e., distressing, situations. The causal-temporal event chain of the secure base script consists of three main blocks: when a child encounters a stressor, (1) (s)he signals for or seeks help from the caregiver; (2) the caregiver is available, is responsive and provides support; and (3) the child experiences stress relief and can resume normal functioning. When children repeatedly experience that their caregivers provide effective support during distress, they are proposed to develop secure trait attachment and an easily accessible, generalized secure base script [10,11]. Trait attachment is generally considered a relevant factor for child psychological problems [1]. Specifically, children who are less securely attached at a trait level are more vulnerable to develop psychological problems when experiencing stress, underlain by several mechanisms [12], among which are decreased support-seeking [13] and the use of less adaptive emotion regulation strategies [14].

Interestingly, attachment theory does suggest that there is room for change and trait attachment can be updated in response to changes in the interpersonal environment (‘lawful change’) [15,16]. Bowlby [8] proposed that both stability and change in attachment serve an adaptive function. Stability protects children against short-term fluctuations that average out over the course of the lifespan, due to which they have only limited impact on children’s perception of their relationship and social well-being. The ability to change is important to adjust one’s expectations and interpersonal strategies when the context changes for better or for worse. Research generally shows that trait attachment is moderately stable over time [17] (but see also Groh et al. [18] who reported weak attachment stability from infancy to late adolescence), and that trait attachment stability can be affected by several factors such as parental divorce and, family conflict in childhood and adolescence [19,20] and psychological distress in adulthood [21]. In the approximately ten years before the current publication, researchers have increasingly explored the possibility that attachment also varies on the short-term and incorporated a more flexible, state-like component in their models of attachment [22,23]. The proposition that attachment can best be understood as including a state-like component is supported by empirical studies indicating that within-person variability in attachment expectations exists across contexts [24,25].

### 1.2. State Attachment Variability

In a daily diary study in a middle childhood sample, Bosmans and colleagues [2] assessed state attachment towards one’s mother across days and found considerable variability in state attachment across a period of a week. Additionally, Verhees et al. [4] found that children’s state attachment towards their mother can even vary across situations with similar characteristics, specifically distressing situations. Moreover, the latter study revealed a two-dimensional component structure underlying state attachment variability across distressing situations: a Signal-and-Support component that reflected expectations of support-seeking and -receiving, and a Back-on-Track component that reflected expectations of stress reduction and comfort [4].

Several factors may underlie variability in state attachment. Some factors are context-related, such as momentary attuned or mistuned interactions with the attachment figure [22,23], and experiences of (lack of) support in the context of distress [26]. Additionally, priming studies conducted in adults suggest that brief exposure to secure attachment-related stimuli can evoke a sense of felt security or secure state attachment [24,27]. Other factors are child-related, such as biases in the cognitive processing of attachment-related information [28,29] or differential endocrinologically based responsivity to stress and care [30,31] affecting inter-personal variation in state attachment variability. These factors move the attachment research focus beyond mere sensitive parenting as explanation of children’s (in)secure attachment development. This points to the added value of expanding research on (variability of) state attachment for attachment theory.

A recently proposed model of adult attachment suggests that state attachment fluctuations could be relevant for more general, trait-like attachment development. Specifically, it was suggested that insecure attachment states can be buffered by exposure to a secure context (e.g., an attachment figure who responds in a responsive way to insecure feelings or behavior), and this can enhance trait attachment security over time [22]. Another model proposes that more insecurely attached individuals’ risk for psychopathology can be mitigated by the experience of attachment-related supportive contexts [23]. In line with the latter, priming attachment memories has been found to affect socioemotional functioning in adults and children (e.g., [32,33]). However, at present, it is largely unknown whether such models may also be suitable for describing processes in the development of trait attachment and psychological problems in children. Moreover, it is unclear whether the *degree* to which individuals’ attachment states vary across contexts is relevant at a within-person level, i.e., represents a process related to within-person changes in trait attachment and psychological problems. Some cross-sectional empirical research does point to a between-person association between degree of state attachment variability and (1) trait attachment, and (2) psychological problems [2,4].

### 1.3. Degree of State Attachment Variability

#### 1.3.1. Associations with Trait Attachment

At a between-person level, Bosmans et al. [2] found in their diary studies that children who were more secure on a trait attachment level varied less in their state attachment towards their mother across days. Similarly, children with more trait attachment security seemed to vary less in their state attachment across a variety of distressing situations [4]. (Of note, here, we refer to results by Bosmans et al. [2] and Verhees et al. [4] that were obtained with uncorrected standard deviations (SDs) of state attachment scores as indices of variability.) These findings fit well with the proposition that more securely attached children experience more consistent sensitive care [9]. In addition, research indicates that sensitive mothers are more predictable in their signals [34]. It is therefore likely that children who are more securely attached at a trait level experience their caregivers as more consistent and predictable and thus vary less in their state attachment expectations. Moreover, as mentioned above, based on their experiences more securely attached children are proposed to develop a secure base script about attachment needs that are met [10]. Cognitive scripts such as the secure base script are related to the processing of script-relevant information: information processing is biased in favor of existing script expectations [35]. This means that information that is congruent with existing secure expectations is more likely encoded and processed than information that is incongruent with these expectations. These information processing biases increase the likelihood that securely attached children’s subjective experience of care-related interactions with the attachment figure confirms their existing expectations [28,29]. Information that is incongruent with current expectations should become assimilated to existing expectations. Stated differently, for more securely attached children, contextual factors are less likely to result in substantial state attachment deviations from their overall attachment expectations [2].

Children who are more insecurely attached at a trait level may have experienced less predictable and sensitive caregiving [9]. It was proposed that these children develop a certain cognitive structure based on their attachment experiences, i.e., an internal working model [8], More insecure internal working models may contain representations of the caregiver as inconsistent or unpredictable, leading to a higher degree of state attachment variability. Additionally, research shows that insecurely attached children do not develop a secure base script about attachment needs that are met [36]. Rather, a range of alternative schemas were identified in more insecurely attached individuals’ attachment narratives that seem organized more thematic than script-like [37]. The lack of a cognitive script around stress and support may further affect the stability of state attachment at the level of expectations of trust in maternal support. That is, for more insecurely attached children, contextual cues might relate to their expectations of trust in maternal support more strongly than for children who do have a secure base script, resulting in negative between-level associations between attachment security and degree of state attachment variability.

These associations may also be relevant at a within-person level. That is, the development of secure trait attachment (a secure base script) may lead children to increasingly process information in line with their secure base script, leading to intra-individual decreases in state attachment variability. On the other hand, an effect in the opposite direction could also be predicted: children who start to vary less in their attachment states may be developing secure trait attachment [8,38]. Examination of associations on a within-person level could clarify whether these are relevant to understand intra-individual changes, and if so, whether secure trait attachment is a determinant or consequence of less variability in state attachment.

#### 1.3.2. Associations with Psychological Problems

Studies have been scarce, but some research suggests that there is a between-person level association between degree of state attachment variability and well-being. In adults, higher degree of variability in attachment towards the romantic partner was associated with stronger declines in relationship well-being for individuals who were more securely attached at baseline [25]. In children, preliminary research on the association between psychological problems and degree of state attachment variability (tested in the first wave of the current study) suggests that variability is not maladaptive per se. Specifically, Verhees et al. [4] found that higher variability on the Back-on-Track component (reflecting expectations of stress recovery) was concurrently related to higher levels of psychological (internalizing) problems, explaining variance in psychological problems over and above trait attachment measures. Concerning the Signal-and-Support component (reflecting expectations of support-seeking), the results indicated that variability on the Signal-and-Support component was not related to child-reported psychological problems over and above trait attachment, and negatively related to mother-reported child internalizing problems when controlled for trait attachment.

One proposed explanation for degree of (Back-on-Track) state attachment variability being more maladaptive is that variability may represent instability in the appraisal of the caregiver as being able to provide effective support [4,25]. Not being able to ground oneself in the belief that support is effective in relieving distress may increase vulnerability to the negative outcomes of stress, thereby enhancing psychological problems [13]. On the other hand, as abovementioned, variability in the Signal-and-Support component (i.e., variability in expectations of seeking and receiving maternal support) across distressing situations, seems less maladaptive [4]. Being able to flexibly respond to contextual cues, specifically, being able to flexibly assess whether one needs parental support in a particular context may thus reflect a more adaptive kind of variability [4,39]. However, these hypotheses are tentative and the abovementioned results need replication to gain more clarity in the between-level association between degree of state attachment variability and psychological problems. In addition, although one could hypothesize that degree of variability may be a process related to intra-individual variation in psychological problems, this remains unexamined to date.

### 1.4. Current Study

The current study aimed to further explore the associations over time between degree of state attachment variability and (1) trait attachment, and (2) psychological problems by disentangling associations at a between- and within-person level using RI-CLPM [7]. To address this aim, the sample from Verhees et al. [4] (Study 2) was followed up for one year, during which data were collected three times with a six-month interval between each registration period. We focused on state attachment variability across distressing situations. Distressing situations provide an important context for the evaluation of attachment expectations as distress can activate the attachment system and distressing situations are secure base script-relevant. We tested a middle childhood sample (9–12 years old at time 1). In this age period, important changes occur at the level of social, cognitive and biological development [40]. For attachment specifically, research indicates significant secure base script development in middle childhood, making this an interesting period to study processes related to attachment development [41].

The first aim of the present study was to assess (a) whether there is a stable, between-person association between degree of state attachment variability and trait attachment over time, and (b) whether within-person deviations in degree of state attachment variability are linked to within-person deviations in trait attachment over time. Based on reported literature, we predicted a negative between-person association between degree of state attachment variability and trait attachment security. At a within-person level, both directions or a reciprocal relationship could be predicted. The second aim of the study was to explore (a) whether degree of state attachment variability and psychological problems are related in between-persons analyses over time, and (b) whether within-person fluctuations in degree of state attachment variability predict within-person fluctuations in psychological problems or vice versa. Based on previous research in the same sample at time 1 [4], we predicted a positive between-person association between degree of Back-on-Track variability and psychological problems, and no robust between-person association between degree of Signal-and-Support variability and psychological problems. Due to a lack of previous longitudinal studies and a lack of evidence regarding what increases or decreases in state attachment variability may reflect, an important empirical question is whether and if so how such associations are relevant on a within-person level.

## 2. Materials and Methods

### 2.1. Participants

The full sample consisted of 152 children aged 9–12 years at time 1 (T1) and their mothers (same sample as Verhees et al. [4]; Study 2). Participant characteristics are reported in Table 1. At T1, 151 children (99%) and 146 (96%) mothers participated. Follow-up data after six months at time 2 (T2) was present for 148 children (97%) and 136 mothers (89%), and one-year follow-up data at time 3 (T3) was present for 146 children (96%) and 136 mothers (89%). Children who dropped out at T2 and T3 did not differ significantly from those who did not drop out on gender, age or T1 measures of trait attachment, state attachment variability indices or psychological problems in independent samples *t*-tests (*t*s between −1.55 and 1.18, *p*s > 0.17).

### 2.2. Materials

#### 2.2.1. State Attachment Variability

The Secure Base Script Consistency (SBSC) test was administered to assess children’s secure base script-related expectations across distressing situations [4]. The SBSC consists of eight situations describing middle childhood age-appropriate stressors [42], e.g., ‘You are being bullied on the playground by some boys and/or girls. Because of the bullying, you feel sad when you go home’. For every situation, children rated for 18 different scenarios to what extent they expected these to happen on a Likert scale ranging from 1 (*would not happen at all*) to 7 (*would definitely happen*). The 18 scenarios were divided over three blocks, following the three secure base script blocks [10], i.e., expectations concerning (1) seeking of or signaling for maternal support, (2) maternal availability and support, and (3) feeling better afterwards (being ‘back-on-track’). The SBSC questions and answer items (scenarios) can be found in Table 2.

SBSC data were analyzed with multi-level simultaneous component analysis with invariant pattern constraints (MLSCA-P) [43,44], using the software package described in Ceulemans et al. [43]. In line with our research questions, here, we focused on the within-part of the data (the child-specific situational deviations from their own mean scores). Therefore, we person-mean-centered the data before SCA-P analyses. We first performed SCA-P analyses on the SBSC data for the three measurement waves separately. Situations with missing data were not included and participants with less than five situations remaining were excluded from the analyses (this was the case for three participants at T1, one participant at T2 and one participant at T3, who as a result, had missing values on the variability indices for that wave). Per wave, we person-mean-centered the raw data, then scaled data overall and fitted models with 1 to 5 components. The model with two components (obliquely rotated) offered the best balance between amount of variance accounted for and complexity (i.e., number of components) according to the CHull heuristic [45] at every wave, explaining, respectively, 32%, 34% and 33% of the within-person variance in state attachment at T1, T2 and T3. To assess similarity of the components across waves, we calculated Tucker’s coefficients of congruence [46]. These coefficients were 0.99 for the first component and ranged from 0.97 to 0.99 for the second component, indicating that the underlying structure of intra-individual state attachment variability can be considered equal across waves [47].

We then performed a new SCA-P analysis that combined the data of all three waves. This way the components were equal across waves, allowing comparison across waves. The data were centered per person per wave and then scaled overall. Again, we fitted models with 1 to 5 components and the model with two components (obliquely rotated) offered the best fit–complexity balance according to the CHull heuristic. The normalized loadings of this SCA-P analysis can be found in Table 2. The results replicated the component structure that was found in previous research [4] with a Signal-and-Support component and a Back-on-Track component. The correlation between the component scores across children was low (*r* = 0.02). In line with the aims of the current study, we focused on individual differences in how much an individual’s attachment states deviate from their own mean state attachment score across situations. Therefore, we computed per component the standard deviations of component scores per participant across situations and used this as an index of degree of state attachment variability across situations.

#### 2.2.2. Trait Attachment Measures

##### Trust in Maternal Support

Children’s trust in maternal support was measured with the People In My Life (PIML) questionnaire Trust subscale [48]. Children only rated the ten items concerning their mother (e.g., ‘I can count on my mother to help me when I have a problem’). Items were rated on a scale from 1 (*almost never true*) to 4 (*almost always true*). Cronbach’s *α*s were 0.79, 0.85 and 0.90 at T1, T2 and T3, respectively.

##### Anxious and Avoidant Attachment

We measured children’s insecure attachment styles with the abridged version of the Experiences in Close Relationships scale—Revised Child version (brief ECR-RC) [49]. Children rated 12 items concerning the relationship with their mother on a scale from 1 (*strongly disagree*) to 7 (*strongly agree*). Six items measured Attachment anxiety and concerned physical or emotional fear of abandonment (e.g., ‘I’m worried that my mother might want to leave me’). Cronbach’s αs for Attachment anxiety were 0.85 at T1, 0.84 at T2 and 0.91 at T3. Six items measured Attachment avoidance and concerned discomfort with closeness and self-disclosure (e.g., ‘I don’t like telling my mother how I feel deep down inside’). Cronbach’s *α*s for Attachment avoidance were 0.67, 0.80 and 0.82 at T1, T2 and T3, respectively.

##### Secure Base Script Knowledge

Children’s secure base script knowledge was measured with the middle childhood version of the Attachment Script Assessment (ASA) [11]. In this task, children tell stories based on prompt word outlines that contain 12 words, suggesting a beginning, middle and ending of a possible story. Children started with two practice stories about themselves and a friend, followed by three attachment-related stories that revolve around the child and their mother (Scary dog in the yard, At the beach and Soccer game). The prompt words for these stories indicate distress, and the opportunity for mother and child to respond according to the secure base script. The order of administration of the three attachment-related stories was randomly varied across participants. Stories were recorded, transcribed and scored. The scores reflect the amount of secure base script-congruent content present in the story and can range from 1 to 7, with higher scores reflecting more secure base script content.

T1 stories were double-coded by four trained coders. All four coders independently rated ASA stories from the same 30 participants to establish interrater agreement. ICCs (two-way mixed model, absolute agreement for average measures) between the pairs of coders were respectable to excellent (ICCs ranged between 0.72 and 0.94). After establishing interrater agreement, two coders separately rated half of the remaining stories and the other two coders separately rated the other half. All stories were thus double-coded. For most stories (86%), coders differed by less than one point in their score and we used the mean score of the two raters for further analyses. On the remaining 14% of the stories, coders differed by one point or more in their scores, and these stories were discussed until consensus. T2 and T3 stories were single-coded by two trained coders. Coders independently rated the same 20 stories for interrater agreement. ICCs (two-way mixed model, absolute agreement for single measures) were good to excellent at T2 (ICCs ranged from 0.84 to 0.95) and T3 (ICCs between 0.76 and 0.88). After establishing interrater agreement, both coders separately rated half of the remaining stories. The internal consistency for the three stories was acceptable at T1 (*α* = 0.70), T2 (*α* = 0.71) and T3 (*α* = 0.68).

#### 2.2.3. Strengths and Difficulties

The Strengths and Difficulties Questionnaire (SDQ) [50] was administered to children (child version) and their mothers (parent version). The SDQ measures child social and emotional strengths and problems with 25 items (e.g., ‘(I have) many fears, (I am) easily scared’) that are rated on a three-point scale *(not true, somewhat true,* or *certainly true*). The SDQ distinguishes five subscales: emotional problems, conduct problems, hyperactivity/inattention problems, peer problems and prosocial behavior. The four problem scales of the SDQ were combined into a total difficulties score. Cronbach’s αs were acceptable for child-reported total difficulties (T1: *α* = 0.72; T2: *α* = 0.74; T3: *α* = 0.77) and good for mother-reported total difficulties (T1: *α* = 0.81; T2: *α* = 0.80; T3: *α* = 0.80).

### 2.3. Procedure

Participants were recruited by distributing 558 informative letters at elementary schools in Belgium. One hundred fifty-two mothers gave their active informed consent to participate in the three measurement waves (response rate: 27%). Active informed consent was also obtained from the children before the procedure started. Data were collected from children and their mothers three times during one year with a six-month interval between each measurement wave. For all three measurement waves, the procedure for children consisted of a part at school and a part at home. For the current study, we only used the data collected at school. The procedure at school consisted of two parts: a collective part and an individual part. Within each wave, administration order of these two parts was varied (54, 58 and 55% of the participants participated first in the collective part at T1, T2 and T3, respectively). During the collective part, children were seated in a classroom and individually completed the SBSC, PIML Trust, ECR-RC and a social desirability questionnaire (the latter was not used in the present study). Research assistants were present to answer any questions children may have. The collective part lasted 45 min on average. The individual part consisted of the ASA and SDQ and lasted approximately 20 min. As mentioned, at each measurement wave, children also completed a task at home. They filled out a two-week daily diary in which they reported on their state attachment and experiences of maternal support in the context of distress. These diary data were not used in the current study. Children’s mothers completed several online questionnaires during the two weeks children filled out their daily diary. Of these questionnaires, only the SDQ was used for the current study. After each measurement wave, participants received two cinema tickets for their participation. The procedure was approved by the Social and Societal Ethics Committee KU Leuven.

### 2.4. Analytic Strategy

For each of the measures of trait attachment (i.e., Trust, Attachment avoidance, Attachment anxiety and ASA) and psychological problems (i.e., SDQ child report and SDQ mother report), we performed two RI-CLPMs: one with the Signal-and-Support variability index and one with the Back-on-Track variability index. By separating more stable inter-individual differences at a between-person level from within-person effects, RI-CLPMs allow for examining (a) between-person covariation and (b) within-person cross-lagged paths, stability paths and within-time correlations. This way, the within-person paths specifically reflect intra-individual processes [7].

Analyses were performed in Mplus (version 7.31) [51]. We used the robust maximum-likelihood estimator (MLR) to account for non-normality of the data and the full-information maximum-likelihood estimator (FIML) to handle missing data. We followed the procedure outlined in Hamaker et al. [7] for specifying the RI-CLMPs. Random intercepts at the between-level, measured by the observed scores on the three waves, reflect the individual’s trait-like deviations from the grand means across participants. At the within-level, within-person centered scores reflect the individual’s temporal deviations from their own expected scores.

**Figure 1:**
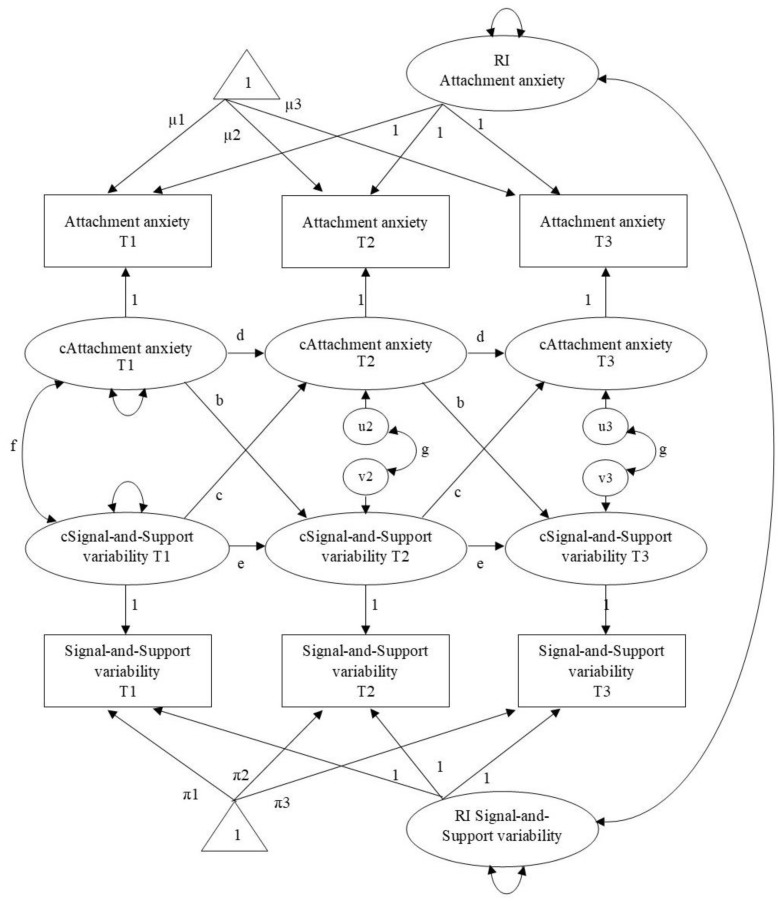
Random Intercept Cross-Lagged Panel Model linking Attachment anxiety with Variability on the Signal-and-Support component, while separating between-person variance from within-person variance. RI = Random intercept; c = within-person centered; µ = grand mean for Attachment anxiety; π = grand mean for Signal-and-Support variability; u = innovation attachment anxiety; v = innovation Signal-and-Support variability.

First, we fitted unconstrained models, i.e., the paths between the random intercepts and between the within-person centered scores were freely estimated. Then, we simplified the models by constraining the within-level parameters to be equal across waves, because (a) the intervals between measurements were of equal length and (b) we assume no differences in developmental processes from T1 to T2 compared with those from T2 to T3 because children differed in age at T1: some children were the same age at T1 as others were at T2. For the models with attachment anxiety, attachment avoidance, ASA, and SDQ child and mother report, the constrained models did not have a worse fit than the unconstrained models based on Satorra–Bentler scaled chi-square difference tests (Δ*S-Bχ*^2^s between 0.18 and 6.80, *p*s between 0.24 and 1). For these measures, we therefore report the constrained models. See Figure 1 for the model for Attachment anxiety and variability on the Signal-and-Support component and Supplementary Material S1 for the Mplus code for this model. Equivalent models were specified for the other measures, except for the models with Trust. The *ΔS-Bχ*^2^s indicated that constraining the within-person parameters led to a significantly worse model fit for the models involving Trust (Signal-and-Support component: Δ*S-Bχ*^2^ = 17.48, *p* < 0.01; Back-on-Track component: Δ*S-Bχ*^2^ = 19.34, *p* < 0.01), suggesting that these parameters could not be considered equal over time. Therefore, no constraints were imposed on these models and within-person parameters were freely estimated.

In post-hoc analyses we explored whether there were group difference in the parameters based on age by running multi-group RI-CLPMs [52]. We split up our sample in two groups based on age at T1 (median split) and ran for all main RI-CLPMs two additional multi-group models: one where no equality constraints across groups were imposed and one were where all parameters were constrained to be equal across groups. We then compared these two models using a Satorra–Bentler scaled chi-square difference test. If this test is not significant, the parameters can be considered similar across groups.

## 3. Results

### 3.1. Preliminary Analyses

For the attachment questionnaires, ASA and SDQs, no total scale scores were calculated when an item was missing. In total, 5% of the data was missing at the scale level. Missing scales were handled in MPlus (FIML). Descriptive statistics and bivariate correlations among the study variables can be found in Supplementary Table S1. We additionally explored whether age was related to the study variables at all timepoints and found only significant positive correlation between age and attachment avoidance at T1, and between age and Back-on-Track variability at T2. None of the other variables at any of the timepoints was significantly associated with age (see Supplementary Table S2). Therefore, we did not control for age in further analyses. We estimated how much variance was due to inter- and intra-individual differences in Mplus by squaring the standardized loadings of the observed scores on the random intercepts. These values reflect the proportion of variance that is accounted for by between-person differences. For each of the variables, a substantial part of the variance was due to between-person differences (ranging between 31 and 80%). However, these estimates also suggest that for all variables, part of the variance (i.e., between 20 and 69%) can be attributed to fluctuations within children.

### 3.2. Degree of State Attachment Variability and Trait Attachment

The final RI-CLPMs for the associations between the trait attachment measures (Trust, Attachment anxiety, Attachment avoidance and ASA) and variability indices (SD Signal-and-Support component and SD Back-on-Track component) showed an acceptable to good fit (see Table 3 and Table 4). The results from the RI-CLPMs for Trust can be found in Table 5 and for Attachment anxiety, Attachment avoidance and ASA in Table 6. In line with our research questions, we focus on the associations between the random intercepts at the between-person level and on the cross-lagged paths at the within-person level.

#### 3.2.1. Trait Trust

**Figure 2:**
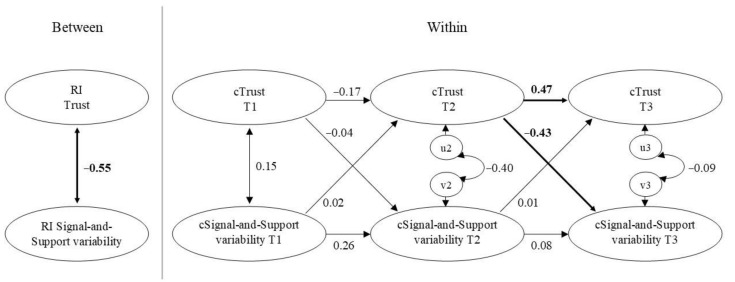
Simplified presentation of the RI-CLPM model between trait Trust and Signal-and-Support variability. The between-level is presented on the left, and the within-level is presented on the right. Standardized estimates are presented. Significant effects are in boldface. RI = Random intercept; c = within-person centered.

At the between-person level, there was a negative relation between variability on the Signal-and-Support component and trait Trust. Children who reported more trust in maternal support over the three measurement waves showed less variability in the Signal-and-Support component over the three waves. There was no between-level association between trait Trust and Back-on-Track variability. At the within-level, deviations in trait Trust at T2 negatively predicted deviations in degree of Signal-and-Support variability at T3: if children’s trait Trust at T2 was higher than their own expected score, this predicted a decrease in Signal-and-Support variability at T3 (relative to their own expected score; see Figure 2). There were no other significant within-person associations between trait Trust and the state attachment variability indices.

#### 3.2.2. Trait Attachment Anxiety and Attachment Avoidance

**Figure 3:**
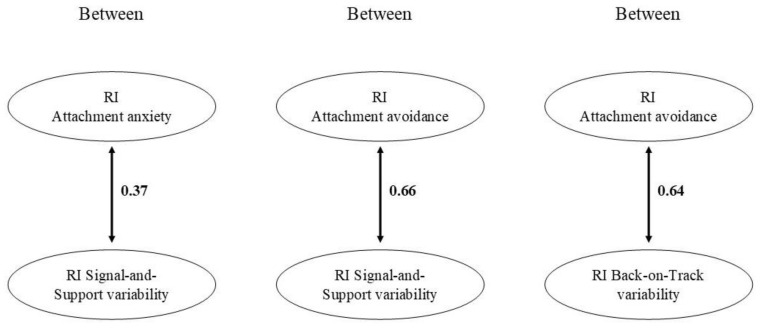
Significant associations from the RI-CLPMs with trait Attachment anxiety and Attachment avoidance. Standardized estimates are presented. RI = Random intercept.

Significant associations are presented in Figure 3. At the between-person level, there was a positive relation between variability on the Signal-and-Support component and both trait Attachment anxiety and Attachment avoidance. Children who were more anxiously or avoidantly attached over the three measurement waves showed more variability in the Signal-and-Support component over the three waves. Moreover, trait Attachment avoidance was positively associated with Back-on-Track variability: children who were more avoidantly attached across the three waves varied more in their Back-on-Track expectations across waves. At the within-level, there were no significant cross-lagged paths between trait Attachment anxiety and either of the variability indices or between trait Attachment avoidance and the variability indices.

#### 3.2.3. Secure Base Script Knowledge

There were no significant associations between secure base script knowledge and the variability indices, neither at the between-person level nor at the within-person level.

### 3.3. Degree of State Attachment Variability and Psychological Problems

The RI-CLPMs for the associations between psychological problems (SDQ child and mother report) and the variability indices showed a good fit (Table 4). The results from the RI-CLPMs are presented in Table 7. Again, we focus on the associations between the random intercepts at the between-person level and on the cross-lagged paths at the within-person level.

#### 3.3.1. Child-Reported Psychological Problems

**Figure 4:**
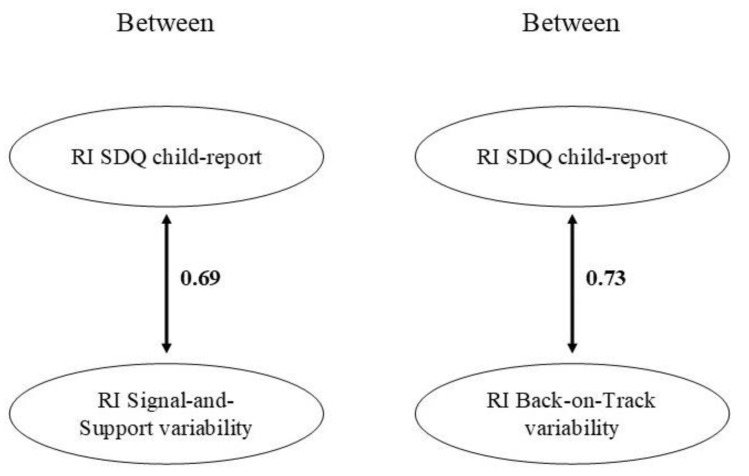
Significant associations from the RI-CLPMs with SDQ child-report. Standardized estimates are presented. RI = Random intercept; SDQ = Strengths and Difficulties Questionnaire, total problems scale.

Significant associations are presented in Figure 4. At the between-person level, we found that child-reported psychological problems were positively linked to variability on both the Signal-and-Support and the Back-on-Track component. Children who overall reported more psychological problems overall varied more in their state attachment across distressing situations. (When we distinguished between internalizing problems and externalizing problems in the SDQ [53], both variability indices were positively linked to both internalizing and externalizing problems at a between-person level.) At the within-level, there were no significant associations between child-reported psychological problems and the state attachment variability indices.

#### 3.3.2. Mother-Reported Psychological Problems

**Figure 5:**
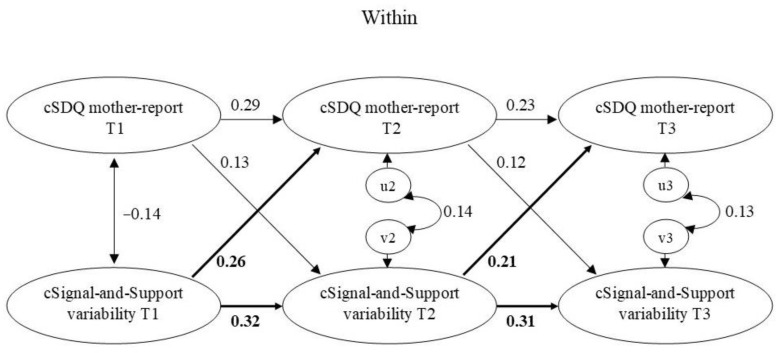
Within-level model for SDQ mother report and Signal-and-Support variability. Standardized estimates are presented. Significant effects are in boldface. c = within-person centered; SDQ = Strengths and Difficulties Questionnaire, total problems scale.

No significant between-person associations were found between mother-reported psychological problems and the variability indices. At the within-person level, the cross-path from Signal-and-Support variability to mother-reported psychological problems was significant. Variability on the Signal-and-Support component positively predicted mother-reported problems, indicating that children who positively deviated from their own expected score on Signal-and-Support variability showed more psychological problems according to their mother six months later (see Figure 5). (When we distinguished between internalizing problems and externalizing problems, analyses showed that this finding was specific for externalizing problems: there was a cross-lagged effect from Signal-and-Support variability to mother-reported externalizing problems and not to mother-reported internalizing problems.) There were no other significant within-person associations between mother-reported psychological problems and the state attachment variability indices.

### 3.4. Multi-Group Models Based on Age

Comparison of multi-group RI-CLPMs revealed that constraining parameters to be equal across age groups did not result in worse fitting models for the RI-CLPMs with attachment anxiety, attachment avoidance, ASA, and SDQ mother-report (Δ*S-Bχ*^2^s between 1.72 and 12.30, *p*s between 0.09 and 0.98). This indicates that parameters in these models were similar across age groups. For the models with trust and SDQ child-report, we could not compare the models with unconstrained and constrained parameters across groups as the former models would not converge or generated warnings. Likely these models were too complex for our data due to our limited sample size.

## 4. Discussion

The current study aimed to disentangle between-person and within-person associations over time between degree of state attachment variability and (1) trait attachment, and (2) psychological problems. The results revealed that the degree of state attachment variability was mainly relevant to understand differences *between* children in terms of trait attachment and psychological problems, such that children who overall showed more state attachment variability were overall less securely attached at a trait-level and reported higher levels of psychological problems. Evidence for within-person associations was less robust, with only two significant cross-lagged effects: intra-individual elevations in Trust at T2 predicted intra-individual decreases in Signal-and-Support variability at T3 and intra-individual elevations in Signal-and-Support variability predicted elevated mother-reported psychological problems six months later. Supported by advanced statistical tools, the current study provides a new and unique approach to the examination of the (within- and between-person) correlates of the degree of state attachment variability.

### 4.1. Degree of State Attachment Variability and Trait Attachment

At a between-level, the current results indicated that children who had more self-reported secure trait attachment over the three measurement waves showed less state attachment variability overall (in specific Signal-and-Support variability). This finding largely replicates previous cross-sectional studies in which children who scored higher on (self-reported) secure trait attachment seemed to vary less in their state attachment across days [2] and across distressing situations [4]. While in the latter study both variability in expectations of support-seeking and –receiving, and variability in expectations of getting back-on-track after distress were significantly related to trait attachment, in the current study, only Signal-and-Support variability was robustly associated with different self-reported trait attachment measures. Back-on-Track variability was only associated with attachment avoidance. A post hoc explanation for these findings could be that the Signal-and-Support component as operationalized in the current study might tap more into the core of the secure base script and secure attachment, as compared with the Back-on-Track component. Specifically, while resuming normal functioning after distress (getting back-on-track) would be at the core of attachment when it reflects repair in a dyadic fashion, the non-significant correlations between scores on the Signal-and-Support component and Back-on-Track component indicate that, in this case, resuming normal functioning after distress was not necessarily related to maternal support. Therefore, the Back-on-Track component might not capture repair in a dyadic fashion but rather children’s autonomous recovery.

Based on the importance of consistent caregiver sensitivity for trait attachment security [9] and on the secure base script hypothesis [10] and attachment information processing theory [28], one would indeed expect that secure trait attachment is related to less substantial variability in attachment-relevant expectations specifically. That is, more securely attached children have likely experienced more predictable and sensitive caregiving due to which they vary less in their state attachment expectations. In contrast, more insecurely attached children may experience less predictable and sensitive caregiving and their representations of the caregiver as more inconsistently sensitive may lead to higher variability in state attachment expectations. Moreover, more securely attached children are proposed to develop a secure base script that biases the processing of attachment-related information and increases the likelihood that script-incongruent information is assimilated with their existing expectations, which should result in even less variability in attachment states. Of note, there were no significant associations between the variability indices and secure base script knowledge as measured with the ASA in the current study. This is a replication of previous findings with (uncorrected) variability indices in middle childhood [4]. Nonetheless, these findings are not in line with the abovementioned theoretical proposition that having knowledge of and access to the secure base script should relate to less state attachment variability.

One potential explanation for the absence of associations between degree of state attachment variability and secure base script knowledge between children is that children who do not develop a secure base script may develop other cognitive schemas, such as insecure internal working models [8] or early maladaptive schemas [54,55]. Such schemas can also bias information processing, resulting in less variability in attachment states. In line with this suggestion, insecure attachment processing biases have been reported in the literature [28,29]. However, one could argue that the robust associations between the self-reported trait attachment measures and the degree of state attachment variability found in the current study speak against the explanation that the lack of associations between ASA and the variability indices were due to insecure attachment, and related schemas and information processing biases. Moreover, in adults, no specific anxious or avoidant scripts have been identified in more insecurely attached individual’s attachment narratives, but rather, a range of alternative schemas that did not seem organized in a script-like fashion [37].

Other explanations for the absence of between-person associations between state variability and ASA scores may exist. For instance, a recent study found that the secure base script considerably develops in middle childhood, with significant increases in secure base script knowledge from middle childhood to early adolescence [41]. In line with these findings, in the current study average ASA scores also significantly increased from T1 (*M* = 3.66, *SD* = 0.59) to T3 (*M* = 3.93, *SD* = 0.59). Nonetheless, at T3, still only 45% of the children had (at least some) secure base script knowledge based on the ASA scoring principles (i.e., a score of 4 or higher indicates the presence of secure base script knowledge). One could hypothesize that different attachment-related cognitive constructs (e.g., secure base script, conscious appraisals of attachment security, information processing biases and assimilation) develop at different paces. It might be that secure attachment-related information processing biases and assimilation are already at play before secure base script knowledge shows in the ASA. This may have limited our ability to find relationships at the between-person level between state attachment variability indices and secure base script knowledge.

Interesting in this matter is that there was no robust evidence for any within-person associations of degree of state attachment variability with secure base script knowledge, or with self-reported trait attachment measures in the current study. Only the negative cross-lagged effect from Trust at T2 to Signal-and-Support variability at T3 was significant, which indicated that children who more positively deviated from their expected Trust score at T2 varied less than expected in Signal-and-Support variability at T3. However, since eight different models with trait attachment and state attachment variability indices were tested, and only this within-person association was found, this might reflect a type 1 error. Therefore, we should be cautious interpreting this finding. The lack of significant within-person associations suggests that there was no clear developmental within-person relationship between degree of state attachment variability and trait attachment in the current sample for the duration of the study. One could take this as an indication that secure trait attachment is not a clear determinant or consequence of lower levels of state attachment variability.

Nonetheless, alternative explanations for the absence of such relationships are possible. For instance, a lack of power may have complicated finding significant within-person associations. Specifically, larger samples or more measurement waves may be needed to be able to adequately test whether associations at a within-person level exist. Additionally, the spacing of the assessments may have not been ideal to capture developmental processes in the currently measured constructs. For instance, the time frame of one year may have been too short and follow-ups into an older age may be needed to allow for changes in trait attachment to occur. The latter proposition is endorsed by the current sample’s ASA scores, which indicate that most children had not (yet) developed secure base script knowledge at T3.

### 4.2. Degree of State Attachment Variability and Psychological Problems

At a between-level, we found associations between degree of state attachment variability and self-reported psychological problems: children who generally reported more psychological problems overall varied more in their state attachment (on both components) across distressing situations. Replicating the cross-sectional results that we reported previously about the first wave of data in this sample [4], we also found longitudinally that higher Back-on-Track variability was associated with more psychological problems between children. In the current study, higher Signal-and-Support variability was also related to more self-reported psychological problems, which was not predicted based on the previous cross-sectional findings in this sample [4]. Of note, contrary to the cross-sectional study, in the current study, we did not control for trait attachment for estimation as well as power reasons, i.e., the current sample size was too small to allow for adding covariates into the complex RI-CLPMs. Thus, here, we did not test whether, at a between-person level, degree of state attachment variability is associated with psychological problems over and above trait attachment. This remains to be tested in studies in which a larger sample size allows for controlling for trait attachment measures in RI-CLPM analyses. There were no between-level associations between mother-reported child psychological problems and degree of state attachment variability, which was unexpected based on the cross-sectional associations found in the first wave of data in this sample [4]. However, the current within-level results suggested that Signal-and-Support variability was relevant in the prediction of mother-reported child problems six months later.

Specifically, children who positively deviated from their own expected score on Signal-and-Support variability showed more psychological problems according to their mother at the next measurement wave. This finding suggests that state attachment variability may reflect a process through which children become more vulnerable to the development of psychological problems. This raises the question why increased instability regarding whether one expects to seek and receive support in different distressing situations may be maladaptive. One explanation could be that, in the current community sample, increased Signal-and-Support variability reflects that children are less able to ground themselves in the belief that, upon distress, they can seek maternal support and their mother will be willing and available to provide such support. This may lead children to deferring actual support-seeking, which can increase their vulnerability to distress and result in increased psychological problems six months later [13]. However, since this was the only significant within-person effect found in the models with psychological problems, further research is needed to test the robustness of this finding.

### 4.3. Limitations, Future Directions and Implications

In the current study, we used uncorrected SDs as indices of state attachment variability. The debate on how to quantify degree of variability is ongoing, as an individual’s SD is interconnected with this individual’s mean score across measurements [56,57]. Therefore, the currently used uncorrected SDs contain, besides variability information, information about the mean. A possible solution to this problem is to add the mean score as a control variable in the analyses. However, it has been questioned whether such an approach is superior as, (1) if one conceptually expects the mean and variability to be related, one might be filtering out meaningful variance [56] and (2) interpretation of the association between variability and another variable of interest may be complicated, especially in case of multicollinearity (i.e., in practice, it does not make sense to assume that variability will change while the mean is held constant if these are interconnected [57]). Moreover, in the current study, a lack of power prohibited us from running the RI-CLPMs with mean state attachment scores as a covariate. Studies that include a larger sample are needed to allow such analyses.

Another solution is to use relative variability indices that account for the extent to which individuals can vary given their mean score [57]. We previously used relative SDs in the examination of cross-sectional correlates of degree of state attachment variability, and results with these relative SDs diverged from those with uncorrected SDs [4]. Where results with the uncorrected SDs indicated that children with more secure trait attachment overall did not vary substantially in their state attachment, results with the relative SDs suggested that these children are not rigid and remain flexible in response to contextual fluctuations [4]. In the current study’s main analyses, we focused on the uncorrected SDs, because interpretation of the RI-CLPM findings with relative SDs is challenging, as both between- and within-person levels are involved. Nevertheless, we also ran RI-CLPMs including relative SDs as variability indices to check whether results diverged from those with the uncorrected SDs. The results from these analyses and preliminary interpretations can be found in Supplementary Material S2.

The current study was limited because most of the significant associations were between child self-reported measures, which can lead to the concern that results mainly reflect reporter bias. Nevertheless, we did also find a relation between variability in child-reported attachment states and mother-reported psychological problems. Moreover, we did not measure actual experiences of distress or actual attachment-related behavior. The current study’s focus on expectations warrants the use of the self-reported SBSC, as it might be difficult to provide insight into attachment expectations with for instance observational measures [58]. Nevertheless, future research could investigate whether the current findings replicate with other variability measures, such as story-telling about distressing situations or ambulatory measures.

In the current study, we tested a community sample of children who, overall, showed high levels of trust and low levels of attachment anxiety and attachment avoidance. Future research could consider testing the current research questions in a more insecurely attached sample as this could allow testing whether children who are more insecurely attached might also show low variability due to insecure attachment-related information processing biases. Additionally, previous adult research suggested that variability may be specifically maladaptive for individuals who are more securely attached at the trait level [25], and testing the association between degree of state attachment variability and psychological problems in a more heterogeneous sample in terms of attachment security may shed light on whether these dynamics serve the same function in middle childhood. A final limitation of this study is that we only focused on the mother–child attachment relationship. Future studies may examine associations of state attachment variability with trait attachment and psychological problems within other attachment relationships such as between father and child to see whether findings converge.

Despite the abovementioned limitations, the current study has some implications for attachment theory and clinical practice. First, in line with previous studies (e.g., [2,4]), we found that children vary in their attachment expectations across distressing situations. This suggests that contextual factors can affect children’s attachment expectations and provides further evidence that attachment should not be solely considered a stable construct but rather that at-the-moment state attachment expectations can vary by context. Therefore, models of attachment may benefit from incorporating state attachment besides trait attachment. Second, the degree of state attachment variability across distressing situations seems an inter-individual difference factor related to trait attachment. As mentioned above, this may be due to secure attachment-related information biases for more securely attached children. The present study did not provide indications that within-person changes in state attachment variability without any training or intervention are linked to development of trait attachment. Nevertheless, it seems interesting for clinical interventions that aim to enhance secure trait attachment to test whether it would be valuable to target, besides the caregiving context, secure attachment-related biases that may decrease variability in secure state attachment [59]. Third, aiming to reduce state attachment variability may have positive effects on children’s psychological problems as we found that lower variability in state attachment was related to lower levels of psychological problems. Especially interesting in this regard was the indication that decreasing children’s state attachment variability may decrease their chance of developing psychological problems. Thus, reducing state attachment variability may be a target for clinical interventions. Of note, the current study cannot infer causality, and therefore, it remains to be tested whether decreasing state attachment variability through intervention can indeed reduce children’s psychological problems.

### 4.4. Conclusions

The current study contributes to our understanding of the interplay between state attachment, trait attachment and psychological problems in middle childhood over time and provides a new and unique approach to examine these associations at between- and within-person levels. The current study suggests that the degree of state attachment variability across distressing situations is an individual difference factor relevant for understanding differences *between* children in trait attachment and psychological problems over time. Specifically, higher intra-individual variability in state attachment was associated with less secure trait attachment and more psychological problems at a between-person level. In addition, there was some indication that degree of state attachment variability might reflect a factor that is relevant for the development of psychological problems at a within-person level. As most robust associations were found at the between-person level, it seems that the associations between these differences are mostly characterized by time-invariant individual differences, at least across the currently measured time frame and number of measurements.

## Figures and Tables

**Table 1:** Participant characteristics at time 1.

Variable		Mean (SD)/Number (%)
Age		10.41 (0.60)
Gender	Boy	55 (36%)
	Girl	97 (64%)
Nationality	Belgium	135 (89%)
	Other	9 (6%)
Living situation	Cohabitating parents	112 (74%)
	One-parent household	23 (15%)
	Blended family	8 (5%)
	Adoptive family	3 (2%)
Maternal educational level	Elementary school or high school	59 (39%)
	Bachelor degree	63 (41%)
	Master degree	23 (15%)
Paternal educational level	Elementary school or high school	67 (44%)
	Bachelor degree	29 (19%)
	Master degree	22 (15%)

Note. Missing data: 5% children’s nationality; 4% children’s living situation; 5% maternal educational level; 22% paternal educational level.

**Table 2:** Normalized SCA-P Loadings.

SBS Block & Question	Answer Item	Component 1 (Signal-and-Support)	Component 2 (Back-on-Track)
1. What would you do in this situation?	I go to my mom.	0.69	0.07
	I let my mom know that I am not okay.	0.68	−0.12
	I resolve it on my own.	−0.66	0.07
	I do not do anything.	−0.36	0.04
	I tell my mom.	0.73	0.07
	I keep my feelings to myself.	−0.54	−0.07
2. What would your mom do in this situation?	Mom is too busy with other things.	−0.38	−0.05
	Mom says it is my own problem and I have to learn to deal with it by myself.	−0.35	−0.05
	Mom tells me that everything will be okay.	0.45	−0.02
	Mom gives advice on how I can handle it.	0.45	−0.10
	Mom tries to resolve it for me.	0.45	0.04
	Mom does not notice anything.	−0.42	0.03
3. How do you feel afterwards?	I still do not know what to do.	0.00	−0.46
	I feel happy again.	0.01	0.75
	Thanks to my mom, I do not worry anymore.	0.18	0.59
	It is easier to do something else again.	0.02	0.72
	I keep worrying about it.	0.13	−0.67
	I feel alone.	0.05	−0.55

Note. SBS = secure base script.

**Table 3:** Model fit indices for RI-CLPMs with Trust with freely estimated (unconstrained) within-person parameters.

Model		*χ*^2^ (1)		RMSEA	CFI	TLI	SRMR
x	y (Variability Index)		*p*				
Trust	Signal-and-Support	0.00	0.96	0.00	1.00	1.04	0.00
	Back-on-Track	0.75	0.39	0.00	1.00	1.01	0.01

Note. RMSEA = root mean square error of approximation; CFI = comparative fit index; TLI = Tucker– Lewis Index; SRMR = standardized root mean square residual.

**Table 4:** Model fit indices for RI-CLPMs with constrained within-level parameters.

Model		*χ*^2^ (6)		RMSEA	CFI	TLI	SRMR
x	y (Variability Index)		*p*				
Attachment anxiety	Signal-and-Support	2.39	0.88	0.00	1.00	1.05	0.03
	Back-on-Track	1.64	0.95	0.00	1.00	1.07	0.02
Attachment avoidance	Signal-and-Support	7.62	0.27	0.04	1.00	0.99	0.03
	Back-on-Track	6.48	0.37	0.02	1.00	1.00	0.03
ASA	Signal-and-Support	4.26	0.64	0.00	1.00	1.02	0.03
	Back-on-Track	5.21	0.52	0.00	1.00	1.01	0.04
Psychological problems:	Signal-and-Support	6.70	0.35	0.03	1.00	1.00	0.04
child report	Back-on-Track	1.10	0.98	0.00	1.00	1.04	0.01
Psychological problems:	Signal-and-Support	2.24	0.90	0.00	1.00	1.02	0.02
mother report	Back-on-Track	0.28	1.00	0.00	1.00	1.04	0.01

Note. ASA = Attachment Script Assessment; RMSEA = root mean square error of approximation; CFI = comparative fit index; TLI = Tucker– Lewis Index; SRMR = standardized root mean square residual.

**Table 5:** RI-CLPM results with Trust.

		Between	Within										
		RIx 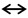 RIy	cx1 → cy2	cx2 → cy3	cy1 → cx2	cy2 → cx3	cx1 → cx2	cx2 → cx3	cy1 → cy2	cy2 → cy3	cx1 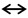 cy1	u2 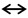 v2	u3 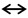 v3
x	y (Variability Index)	(a)											
Trust	Signal-and-Support	−0.07 *	−0.10	−0.75 *	0.01	0.01	−0.20	0.71 **	0.24	0.07	0.01	−0.03	−0.01
	Back-on-Track	−0.04	−0.88	−0.36x	−0.13	−0.05	−0.15	0.70 **	0.21	0.21	−0.02	−0.04	−0.02

Note. Unstandardized parameters are reported. RI = random intercept; c = within-person centered; u = innovation x; v = innovation y. * *p* < 0.05. ** *p* < 0.01.

**Table 6:** RI-CLPM results with Attachment anxiety, Attachment avoidance and ASA.

		Between	Within					
		RIxx 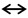 RIy	cx → cy	cy → cx	cx → cx	cy → cy	cx1 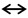 x cy1	u 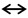 v
x	y (Variability Index)	(a)	(b)	(c)	(d)	(e)	(f)	(g)
Attachment anxiety	Signal-and-Support	0.10 *	0.02	0.03	-0.06	0.29 *	0.05	0.04
	Back-on-Track	0.09	0.05	0.17	-0.07	0.27	0.09 *	0.04
Attachment avoidance	Signal-and-Support	0.18 *	0.07	0.35	0.36 *	0.18	0.08	0.11 **
	Back-on-Track	0.16 *	0.04	0.23	0.43 *	0.21	0.06	0.04
ASA	Signal-and-Support	0.01	0.11	0.16	0.18	0.27	0.02	0.03
	Back-on-Track	0.00	0.07	0.20	0.10	0.24	0.02	0.02

Note. Unstandardized parameters are reported. RI = random intercept; c = within-person centered; u = innovation x; v = innovation y; ASA = Attachment Script Assessment. * *p* < 0.05. ** *p* < 0.01.

**Table 7:** RI-CLPM results with Psychological problems.

		Between	Within					
		RIx 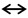 x RIy	cx → cy	cy → cx	cx → cx	cy → cy	cx1 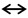 x cy1	u 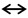 x v
x	y (Variability Index)	(a)	(b)	(c)	(d)	(e)	(f)	(g)
Psychological problems: child report	Signal-and-Support	0.97 **	−0.02	−0.53	0.31 *	0.35 **	−0.20	0.16
	Back-on-Track	1.06 ***	−0.02	−0.41	0.22	0.28	−0.03	0.10
Psychological problems: mother report	Signal-and-Support	0.05	0.02	1.44 *	0.26	0.29 *	−0.17	0.12
	Back-on-Track	0.51	0.01	0.80	0.22	0.26	0.08	0.12

*Note.* Unstandardized parameters are reported. RI = random intercept; c = within-person centered; u = innovation x; v = innovation y. * *p* < 0.05. ** *p* < 0.01. *** *p* < 0.001.

## Data Availability

The data presented in this study are available from the corresponding author upon reasonable request.

## Supplementary Materials

The following are available online at https://www.mdpi.com/article/10.3390/brainsci11101264/s1, Supplemental Material S1: Mplus code RI-CLPM; Table S1: Table reporting descriptive statistics of and correlations among the study’s key variables; Table S2: Table reporting correlations between the study’s key variables and age per assessment time; Supplemental Material S2: RI-CLPMs with relative SDs as variability indices.

## References

[1] Groh A.M., Fearon R., van Ijzendoorn M., Bakermans-Kranenburg M.J., Roisman G.I. (2016). Attachment in the early life course: Meta-analytic evidence for its role in socioemotional development. Child Dev. Perspect..

[2] Bosmans G., Van De Walle M., Goossens L., Ceulemans E. (2014). (In)variability of attachment in middle childhood: Secure base script evidence in diary data. Behav. Chang..

[3] Fraley R.C. (2007). A connectionist approach to the organization and continuity of working models of attachment. J. Pers..

[4] Verhees M.W.F.T., Ceulemans E., Ijzendoorn M.H., Bakermans-Kranenburg M.J., Bosmans G. (2019). State attachment variability across distressing situations in middle childhood. Soc. Dev..

[5] Molenaar P.C., Campbell C.G. (2009). The new person-specific paradigm in psychology. Curr. Dir. Psychol. Sci..

[6] Hamaker E.L. (2012). Why researchers should think “within-person”: A paradigmatic rationale. Handbook of Research Methods for Studying Daily Life.

[7] Hamaker E.L., Kuiper R.M., Grasman R.P.P.P. (2015). A critique of the cross-lagged panel model. Psychol. Methods.

[8] Bowlby J. (1969). Attachment. Attachment and Loss.

[9] Ainsworth M.D.S., Blehar M.C., Waters E., Wall S. (1978). Patterns of Attachment.

[10] Waters H.S., Waters E. (2006). The attachment working models concept: Among other things, we build script-like representations of secure base experiences. Attach. Hum. Dev..

[11] Waters T.E.A., Bosmans G., Vandevivere E., Dujardin A., Waters H.S. (2015). Secure base representations in middle childhood across two Western cultures: Associations with parental attachment representations and maternal reports of behavior problems. Dev. Psychol..

[12] Hankin B.L. (2015). Depression from childhood through adolescence: Risk mechanisms across multiple systems and levels of analysis. Curr. Opin. Psychol..

[13] Dujardin A., Santens T., Braet C., De Raedt R., Vos P., Maes B., Bosmans G. (2016). Middle childhood support-seeking behavior during stress: Links with Self-Reported Attachment and Future Depressive Symptoms. Child Dev..

[14] Verhees M.W.F.T., Finet C., Vandesande S., Bastin M., Bijttebier P., Bodner N., Van Aswegen T., Van de Walle M., Bosmans G. (2021). Attachment and the development of depressive symptoms in adolescence: The role of regulating positive and negative affect. J. Youth Adolesc..

[15] Bowlby J. (1973). Separation. Attachment and Loss.

[16] Sroufe L.A., Coffino B., Carlson E.A. (2010). Conceptualizing the role of early experience: Lessons from the Minnesota longitudinal study. Dev. Rev..

[17] Fraley R.C. (2002). Attachment stability from infancy to adulthood: Meta-analysis and dynamic modeling of developmental mechanisms. Pers. Soc. Psychol. Rev..

[18] Groh A.M., Roisman G.I., Booth-LaForce C., Fraley R.C., Owen M.T., Cox M.J., Burchinal M.R. (2014). Stability of attachment security from infancy to late adolescence. Monogr. Soc. Res. Child Dev..

[19] Jones J.D., Fraley R.C., Ehrlich K.B., Stern J.A., Lejuez C., Shaver P.R., Cassidy J. (2017). Stability of attachment style in adolescence: An empirical test of alternative developmental processes. Child Dev..

[20] Waters T.E.A., Yang R., Finet C., Verhees M.W.F.T., Bosmans G. (2021). An empirical test of prototype and revisionist models of attachment stability and change from middle childhood to adolescence: A 6-year longitudinal study. Child Dev..

[21] Stern J.A., Fraley R.C., Jones J.D., Gross J.T., Shaver P.R., Cassidy J. (2018). Developmental processes across the first two years of parenthood: Stability and change in adult attachment style. Dev. Psychol..

[22] Arriaga X.B., Kumashiro M., Simpson J., Overall N.C. (2017). Revising working models across time: Relationship situations that enhance attachment security. Pers. Soc. Psychol. Rev..

[23] Kobak R., Bosmans G. (2019). Attachment and psychopathology: A dynamic model of the insecure cycle. Curr. Opin. Psychol..

[24] Gillath O., Hart J., Noftle E.E., Stockdale G.D. (2009). Development and validation of a state adult attachment measure (SAAM). J. Res. Pers..

[25] Girme Y.U., Agnew C.R., VanderDrift L.E., Harvey S.M., Rholes W.S., Simpson J.A. (2018). The ebbs and flows of attachment: Within-person variation in attachment undermine secure individuals’ relationship wellbeing across time. J. Pers. Soc. Psychol..

[26] Vandevivere E., Bosmans G., Roels S., Dujardin A., Braet C. (2017). State trust in middle childhood: An experimental manipulation of maternal support. J. Child Fam. Stud..

[27] Bosmans G., Bowles D.P., Dewitte M., De Winter S., Braet C. (2014). An experimental evaluation of the state adult attachment measure: The influence of attachment primes on the content of state attachment representations. J. Exp. Psychopathol..

[28] Dykas M.J., Cassidy J. (2011). Attachment and the processing of social information across the life span: Theory and evidence. Psychol. Bull..

[29] Zimmermann P., Iwanski A. (2015). Attachment in middle childhood: Associations with information processing. New Dir. Child Adolesc. Dev..

[30] Bosmans G., Bakermans-Kranenburg M.J., Vervliet B., Verhees M.W., van Ijzendoorn M.H. (2020). A learning theory of attachment: Unraveling the black box of attachment development. Neurosci. Biobehav. Rev..

[31] Houbrechts M., Cuyvers B., Goossens L., Bijttebier P., Bröhl A.S., Calders F., Chubar V., Claes S., Geukens F., Van Leeuwen K. (2021). Parental support and insecure attachment development: The cortisol stress response as a moderator. Attach. Hum. Dev..

[32] Gillath O., Karantzas G. (2019). Attachment security priming: A systematic review. Curr. Opin. Psychol..

[33] Stupica B., Brett B.E., Woodhouse S.S., Cassidy J. (2017). Attachment security priming decreases children’s physiological response to threat. Child Dev..

[34] Davis E.P., Stout S.A., Molet J., Vegetabile B., Glynn L.M., Sandman C.A., Heins K., Stern H., Baram T.Z. (2017). Exposure to unpredictable maternal sensory signals influences cognitive development across species. Proc. Natl. Acad. Sci. USA.

[35] Beck A.T. (1964). Thinking and depression: 2. Theory and therapy. Arch. Gen. Psychiatry.

[36] Psouni E., Apetroaia A. (2013). Measuring scripted attachment-related knowledge in middle childhood: The Secure Base Script Test. Attach. Hum. Dev..

[37] Waters T.E.A., Facompré C.R., Waters E., Vaughn B.E., Waters H.S. (2021). Measuring secure base script knowledge in the adult attachment interview. Measuring Attachment: Developmental Assessment across the Lifespan.

[38] De Winter S., Bosmans G., Salemink E. (2016). Exploring the causal effect of interpretation bias on attachment expectations. Child Dev..

[39] La Guardia J.G., Ryan R.M., Couchman C.E., Deci E.L. (2000). Within-person variation in security of attachment: A self-determination theory perspective on attachment, need fulfillment, and well-being. J. Pers. Soc. Psychol..

[40] Del Giudice M. (2015). Attachment in middle childhood: An evolutionary-developmental perspective. New Dir. Child Adolesc. Dev..

[41] Waters T.E.A., Facompré C.R., Van de Walle M., Dujardin A., De Winter S., Heylen J., Santens T., Verhees M., Finet C., Bosmans G. (2019). Stability and change in secure base script knowledge during middle childhood and early adolescence: A 3-year longitudinal study. Dev. Psychol..

[42] Vandevivere E., Braet C., Bosmans G. (2014). Under which conditions do early adolescents need maternal support?. J. Early Adolesc..

[43] Ceulemans E., Wilderjans T., Kiers H.A.L., Timmerman M.E. (2015). MultiLevel simultaneous component analysis: A computational shortcut and software package. Behav. Res. Methods.

[44] Timmerman M.E. (2006). Multilevel component analysis. Br. J. Math. Stat. Psychol..

[45] Ceulemans E., Timmerman M.E., Kiers H.A. (2011). The CHull procedure for selecting among multilevel component solutions. Chemom. Intell. Lab. Syst..

[46] Tucker L.R. (1951). A Method for Synthesis of Factor Analysis Studies.

[47] Lorenzo-Seva U., Berge J.M.F.T. (2006). Tucker’s congruence coefficient as a meaningful index of factor similarity. Methodology.

[48] Ridenour T., Greenberg M.T., Cook E.T. (2006). Structure and validity of people in my life: A self-report measure of attachment in late childhood. J. Youth Adolesc..

[49] Brenning K., Van Petegem S., Vanhalst J., Soenens B. (2014). The psychometric qualities of a short version of the experiences in close relationships scale—Revised child version. Pers. Individ. Differ..

[50] Goodman R. (1997). The strengths and difficulties questionnaire: A research note. J. Child Psychol. Psychiatry.

[51] Muthén L.K., Muthén B.O. (1998). Mplus User’s Guide.

[52] Mulder J.D., Hamaker E.L. (2021). Three Extensions of the Random Intercept Cross-Lagged Panel Model. Struct. Equ. Modeling.

[53] Goodman A., Lamping D.L., Ploubidis G.B. (2010). When to use broader internalising and externalising subscales instead of the hypothesised five subscales on the strengths and difficulties questionnaire (SDQ): Data from British parents, teachers and children. J. Abnorm. Child Psychol..

[54] Bosmans G., Braet C., Van Vlierberghe L. (2009). Attachment and symptoms of psychopathology: Early maladaptive schemas as a cognitive link?. Clin. Psychol. Psychother..

[55] McLean H.R., Bailey H.N., Lumley M.N. (2014). The secure base script: Associated with early maladaptive schemas related to attachment. Psychol. Psychother. Theory Res. Pr..

[56] Baird B.M., Le K., Lucas R.E. (2006). On the nature of intraindividual personality variability: Reliability, validity, and associations with well-being. J. Pers. Soc. Psychol..

[57] Mestdagh M., Pe M., Pestman W., Verdonck S., Kuppens P., Tuerlinckx F. (2018). Sidelining the mean: The relative variability index as a generic mean-corrected variability measure for bounded variables. Psychol. Methods.

[58] Waters H.S., Rodrigues L.M., Ridgeway D. (1998). Cognitive underpinnings of narrative attachment assessment. J. Exp. Child Psychol..

[59] Verhees M.W., Ceulemans E., Bosmans G. (2019). Strengthening attachment-based therapies: A case for cognitive bias modification?. J. Am. Acad. Child Adolesc. Psychiatry.

