# Supplementary material for: State Attachment Variability: Between- and within-Person Level Associations with Trait Attachment and Psychological Problems
Source: Brain Sci. 2021 Sep 24;11(10):1264. doi: 10.3390/brainsci11101264 (PMC8533933; doi:10.3390/brainsci11101264)
Supplement: Supplementary file 1 [file brainsci-11-01264-s001.zip › Supplementary Material S1.pdf]

Supplementary Material S1: Mplus code RI-CLPM.

Mplus code for RI-CLPM with Attachment anxiety and variability on the Signal-and-Support component (within-level parameters constrained to be equal across waves)

```
TITLE:          RI-CLPM Anxious Attachment – Signal-and-Support variability

DATA:           file is Anx_SScSD.dat;

VARIABLE:
NAMES           = ANX1 ANX2 ANX3 SScSD1 SScSD2 SScSD3;
USEVARIABLES    = ANX1 ANX2 ANX3 SScSD1 SScSD2 SScSD3;
MISSING         = all (999);

ANALYSIS:       ESTIMATOR IS MLR;

MODEL:          ! between part of the model
                ! create between factors (random intercepts)
                RIANX by ANX1@1 ANX2@1 ANX3@1;
                RISScSD1 by SScSD1@1 SScSD2@1 SScSD3@1;
                ! Set the residual variances of all FX and FY variables to zero
                ANX1@0 ANX2@0 ANX3@0;
                SScSD1@0 SScSD2@0 SScSD3@0;
                ! within part of the model
                ! create within-person centered variables
                cANX1 BY ANX1@1;
                cANX2 BY ANX2@1;
                cANX3 BY ANX3@1;
                cSScSD1 BY SScSD1@1;
                cSScSD2 BY SScSD2@1;
                cSScSD3 BY SScSD3@1;
                ! Specify the lagged effects between the?
                ! within-person centered latent variables
                cANX2 ON cANX1 cSScSD1 (d c);
                cANX3 ON cANX2 cSScSD2 (d c);
                cSScSD2 ON cSScSD1 cANX1 (e b);
                cSScSD3 ON cSScSD2 cANX2 (e b);
                ! Estimate the correlations within the same wave
                cANX1 WITH cSScSD1(f);
                cANX2 WITH cSScSD2(g);
                cANX3 WITH cSScSD3(g);
                ! Specify that between components are not
                ! correlated with within components at T1
                RIANX WITH cANX1@0 cSScSD1@0;
                RISScSD1 WITH cANX1@0 cSScSD1@0;

OUTPUT:         SAMPSTAT PATTERNS TECH1 TECH4 STDYX;
```
