# Supplementary material for: State Attachment Variability: Between- and within-Person Level Associations with Trait Attachment and Psychological Problems
Source: Brain Sci. 2021 Sep 24;11(10):1264. doi: 10.3390/brainsci11101264 (PMC8533933; doi:10.3390/brainsci11101264)
Supplement: Supplementary file 1 [file brainsci-11-01264-s001.zip › Supplementary Material S2.pdf]

Supplemental Material S2: RI-CLPMs with relative SDs as variability indices.

We also performed the RI-CLPM analyses with relative SDs instead of uncorrected SDs. These relative SDs take into account the maximum possible SD given an individual's mean score (Mestdagh et al., 2018). Because the relative SDs could not be calculated with the components extracted from the component analyses, we created two scales capturing the variable structure of the two components: Scale 1 is the Signal-and-Support scale that is based on the items concerning the first two SBS blocks; Scale 2 is the Back-on-Track scale based on the items from the third SBS block. We first reverse-coded the SBS-incongruent items, and then calculated per person, per situation a mean score of the items of the Signal-and-Support scale and the Back-on-Track scale separately. The relative SDs were calculated by means of the MATLAB code provided by Mestdagh et al. (2018). This was done for both scales separately, such that we had two relative variability indices per person.

For each of the measures of trait attachment (i.e., Trust, Attachment avoidance, Attachment anxiety, ASA) and psychological problems (i.e., SDQ child-report, SDQ mother-report), we performed two RI-CLPMs: one with the Signal-and-Support relative SD and one with the Back-on-Track relative SD. We fitted unconstrained models first. Then we simplified the models by constraining the within-level parameters to be equal across waves. For the models with attachment avoidance, ASA, and SDQ child-and mother-report, and the model with attachment anxiety and Back-on-Track relative SD, the constrained models did not have a worse fit than the unconstrained models based on Satorra–Bentler scaled chi-square difference tests ($\Delta S\text{-}B\chi^2$s between 0.76 and 8.45, *p*s between 0.13 and 0.98). For these measures we therefore report the constrained models. The $\Delta S\text{-}B\chi^2$s indicated that constraining the within-person parameters led to a significantly worse model fit for the models involving Trust and for the model with Attachment anxiety and Signal-and-Support relative SD ($\Delta S\text{-}B\chi^2$s between 13.22 and 20.46, *p*s between .00 and .02), suggesting that these parameters could not be considered equal over time. Therefore, no constraints were imposed on these models and within-person parameters were freely estimated. The final RI-CLPMs with the relative SDs showed an acceptable to good fit (see Table S2.1 and S2.2).

Results from the RI-CLPMs can be found in Table S2.3-S2.5. Generally results differed to some extent from the results with the uncorrected SDs. Specifically, at a between-person level, more trust was related to higher relative variability on both components, similar to what was found in the cross-sectional study (Verhees, Ceulemans, et al., 2019). There were no significant associations between the relative variability indices and attachment avoidance, attachment anxiety, ASA or psychological problems at the between level. At a within-person

level, attachment anxiety and attachment avoidance negatively predicted relative Signal-and-Support variability: elevated insecure trait attachment predicted decreased Signal-and-Support relative variability six months later. Moreover, elevated levels of mother-reported psychological problems (specifically externalizing problems) predicted intra-individual increases in relative Signal-and-Support variability. This suggests that increased externalizing problems according to mother predicts six months later an increase in children's variability of their expectations of seeking-and-receiving maternal support, taken into account how much they can vary given their mean score. Notably, with the uncorrected SDs, we found a cross-path in the different direction, indicating that intra-individual changes in uncorrected Signal-and-Support variability predicted changes in of psychological problems. Although these findings are intriguing, it is difficult to further interpret what the associations with the relative SDs reflect. Specifically, different levels of analysis are involved and we look at a very specific part of state attachment variability (i.e., intra-individual deviations from a person's expected score on relative variability indices across three measurement waves that take into account, at each wave, the maximum variability given their mean score at that wave). Moreover, we had no specific a priori hypotheses regarding the intra-individual relevance of relative degree of state attachment variability for psychological problems. Further statistical dismantling of the associations at different levels is needed to be able to adequately interpret what significant (within-person level) associations actually reflect. In addition, replication of these results is needed to assess how robust the findings are.

Table S2.1

Model fit indices for RI-CLPMs with Trust, and Attachment anxiety and Signal-and-Support variability with unconstrained within-person parameters

Model		χ^2 (1)		RMSEA	CFI	TLI	SRMR
x	y (relative SD)		*p*				
Trust	Signal-and-Support	1.35	.25	.05	0.99	0.98	.03
	Back-on-Track	0.98	.32	.00	1.00	1.00	.02
Attachment anxiety	Signal-and-Support	0.01	.93	.00	1.00	1.13	.00

Note. RMSEA = root mean square error of approximation; CFI = comparative fit index; TLI = Tucker– Lewis Index; SRMR = standardized root mean square residual

Table S2.2

Model fit indices for RI-CLPMs with constrained within-level parameters

Model		χ^2 (6)		RMSEA	CFI	TLI	SRMR
x	y (relative SD)		*p*				
Attachment anxiety	Back-on-Track	0.83	.99	.00	1.00	1.18	.02
Attachment avoidance	Signal-and-Support	8.13	.23	.05	0.99	0.98	.05
	Back-on-Track	9.87	.13	.07	0.98	0.94	.05
ASA	Signal-and-Support	6.26	.39	.02	1.00	1.00	.04
	Back-on-Track	6.35	.39	.02	1.00	0.99	.04
Psychological problems	Signal-and-Support	9.86	.13	.07	0.98	0.96	.05
child-report	Back-on-Track	2.06	.91	.00	1.00	1.06	.02
Psychological problems	Signal-and-Support	5.43	.49	.00	1.00	1.00	.04
mother-report	Back-on-Track	3.47	.75	.00	1.00	1.02	.03

Note. ASA = Attachment Script Assessment; RMSEA = root mean square error of approximation; CFI = comparative fit index; TLI = Tucker– Lewis Index; SRMR = standardized root mean square residual.

Table S2.3

RI-CLPM results for the models with Trust, and the model with Attachment anxiety and relative Signal-and-Support variability

		Between	Within										
		RIx ↔ RIy	cx1 → cy2	cx2 → cy3	cy1 → cx2	cy2 → cx3	cx1 → cx2	cx2 → cx3	cy1 → cy2	cy2 → cy3	cx1 ↔ cy1	u2 ↔ v2	u3 ↔ v3
x	y (relative SD)	(a)											
Trust	Signal-and-Support	0.02**	-0.42	-0.04	-3.00	0.13	-1.59	0.76***	-0.69	0.37*	-0.01	-0.02	0.01**
	Back-on-Track	0.02*	-0.16	0.01	-0.52	-0.02	-0.27	0.74***	0.13	0.12	-0.01	0.00	0.01
Attachment anxiety	Signal-and-Support	0.00	0.02	-0.09*	-1.42	0.13	-0.09	0.10	-0.08	0.35*	-0.01	-0.02	-0.03*

Note. Unstandardized parameters are reported. RI = random intercept; c = within-person centered; u = innovation x; v = innovation y.

**p* < .05. ***p* < .01. ****p* < .001.

Table S2.4

RI-CLPM results for the models with Attachment avoidance, ASA, and the model with Attachment anxiety and relative Back-on-Track variability

		Between	Within					
		RIx ↔ RIy	cx → cy	cy → cx	cx → cx	cy → cy	cx1 ↔ cy1	u ↔ v
x	y (relative SD)	(a)	(b)	(c)	(d)	(e)	(f)	(g)
Attachment anxiety	Back-on-Track	-0.01	0.03	0.18	-0.08	0.16	0.00	-0.01
Attachment avoidance	Signal-and-Support	0.01	-0.04**	-0.72	0.47**	0.35*	-0.02	-0.03**
	Back-on-Track	0.00	-0.02	-0.14	0.43*	0.16	-0.04	-0.03
ASA	Signal-and-Support	0.02	-0.01	-0.04	0.14	0.38*	0.01	0.01
	Back-on-Track	0.01	-0.02	0.05	0.14	0.13	0.00	0.01

Note. Unstandardized parameters are reported. RI = random intercept; c = within-person centered; u = innovation x; v = innovation y; ASA = Attachment Script Assessment.

**p* < .05. ***p* < .01.

Table S2.5

RI-CLPM results with Psychological problems

		Between	Within					
		RIx ↔ RIy	cx → cy	cy → cx	cx → cx	cy → cy	cx1 ↔ cy1	u ↔ v
x	y (relative SD)	(a)	(b)	(c)	(d)	(e)	(f)	(g)
Psychological problems child-report	Signal-and-Support	-0.05	0.00	-1.46	0.19	0.39**	-0.08	-0.05
	Back-on-Track	-0.03	0.00	-1.05	0.18	0.16	-0.07	-0.01
Psychological problems mother-report	Signal-and-Support	-0.16	0.02**	2.37	0.22	0.26	-0.01	0.08
	Back-on-Track	0.04	0.00	-1.26	0.29	0.15	-0.02	-0.01

Note. Unstandardized parameters are reported. RI = random intercept; c = within-person centered; u = innovation x; v = innovation y.

**p < .01.
