# Supplementary material for: State Attachment Variability: Between- and within-Person Level Associations with Trait Attachment and Psychological Problems
Source: Brain Sci. 2021 Sep 24;11(10):1264. doi: 10.3390/brainsci11101264 (PMC8533933; doi:10.3390/brainsci11101264)
Supplement: Supplementary file 1 [file brainsci-11-01264-s001.zip › Supplementary Table S1.pdf]

Table S1: Table reporting descriptive statistics of and correlations among the study's key variables.

	1	2	3	4	5	6	7	8	9	10	11	12	13	14	15	16	17	18	19	20	21	22	23	24
1 T1 Trust	1																							
2 T1 Att. anx.	**-.29**	1																						
3 T1 Att. avo.	**-.53**	**.38**	1																					
4 T1 ASA	.07	-.10	-.06	1																				
5 T1 SDQ$_{child}$	**-.49**	**.32**	**.43**	-.10	1																			
6 T1 SDQ$_{mother}$	-.11	.15	**.19**	-.10	**.40**	1																		
7 T1 SaS var.	**-.29**	**.21**	**.42**	.05	**.25**	-.04	1																	
8 T1 BoT var.	**-.30**	**.27**	**.36**	.09	**.35**	**.19**	**.61**	1																
9 T2 Trust	**.70**	**-.25**	**-.49**	.00	**-.49**	-.06	**-.33**	**-.29**	1															
10 T2 Att. anx.	**-.34**	**.34**	.13	-.10	**.20**	.12	.14	**.21**	**-.40**	1														
11 T2 Att. avo.	**-.52**	**.32**	**.58**	.17	**.36**	.09	**.42**	**.37**	**-.64**	**.28**	1													
12 T2 ASA	.16	-.08	.01	**.51**	-.11	-.01	.04	**.18**	.01	-.18	.08	1												
13 T2 SDQ$_{child}$	**-.39**	**.24**	**.38**	-.03	**.69**	**.42**	**.18**	**.31**	**-.43**	**.23**	**.41**	-.03	1											
14 T2 SDQ$_{mother}$	**-.16**	.14	**.22**	-.09	**.42**	**.84**	.07	**.23**	-.10	**.17**	.12	-.09	**.47**	1										
15 T2 SaS var.	**-.37**	**.22**	**.45**	.15	**.22**	.03	**.64**	**.45**	**-.48**	**.23**	**.54**	**.18**	**.23**	.03	1									
16 T2 BoT var.	**-.35**	**.24**	**.39**	.11	**.30**	**.21**	**.47**	**.60**	**-.37**	**.23**	**.40**	**.18**	**.38**	**.25**	**.56**	1								
17 T3 Trust	**.61**	**-.26**	**-.35**	.00	-.44	-.13	**-.27**	**-.27**	**.78**	**-.45**	**-.49**	.15	**-.40**	-.14	**-.37**	**-.31**	1							
18 T3 Att. anx.	**-.34**	**.35**	.15	-.03	.33	.13	**.20**	.19	**-.38**	**.50**	**.23**	**-.24**	**.30**	.16	**.23**	**.22**	**-.60**	1						
19 T3 Att. avo.	**-.53**	**.31**	**.46**	.14	.40	.10	**.40**	**.34**	**-.62**	**.35**	**.65**	-.06	**.33**	.11	**.45**	**.35**	**-.69**	**.53**	1					
20 T3 ASA	.02	**-.17**	-.06	**.48**	**-.19**	**-.18**	.03	-.03	-.11	-.15	.03	**.55**	-.10	**-.20**	**.19**	.05	-.04	-.12	-.05	1				
21 T3 SDQ$_{child}$	**-.42**	**.21**	**.39**	.04	**.59**	**.41**	**.29**	**.36**	**-.48**	**.22**	**.45**	.03	**.65**	**.43**	**.31**	**.39**	**-.59**	**.44**	**.54**	-.03	1			
22 T3 SDQ$_{mother}$	**-.17**	.12	**.24**	-.17	**.38**	**.78**	.06	.17	-.15	.14	.11	-.12	**.45**	**.83**	.08	**.23**	**-.26**	**.18**	.15	-.16	**.42**	1		
23 T3 SaS var.	**-.35**	**.18**	**.30**	.08	**.29**	.06	**.55**	**.38**	**-.52**	**.20**	**.38**	.11	**.21**	.08	**.67**	**.50**	**-.43**	**.32**	**.48**	.16	**.43**	.15	1	
24 T3 BoT var.	**-.20**	.17	**.24**	.06	**.31**	**.18**	**.31**	**.48**	**-.29**	**.24**	**.26**	.08	**.29**	**.21**	**.30**	**.60**	**-.31**	**.31**	**.29**	.00	**.40**	**.22**	**.43**	1
M	3.62	1.80	2.57	3.66	11.67	8.29	.90	.87	3.67	1.53	2.46	3.79	11.12	7.92	.77	.84	3.63	1.50	2.46	3.93	9.63	8.02	.78	.78
SD	0.36	1.18	1.09	0.59	5.21	5.56	.60	.57	0.38	0.90	1.14	0.60	5.13	5.24	.57	.56	0.44	1.03	1.16	0.59	5.36	5.33	.55	.55

Note. Att. anx. = Attachment anxiety; Att. avo. = Attachment avoidance; ASA = Attachment Script Assessment; SDQ = Strengths and Difficulties Questionnaire. SaS var. = Signal-and-Support variability ; BoT var. = Back-on-Track variability. Correlations significant at $p < .05$ are in boldface.
